# Recombinant Bcl-xL attenuates vascular hyperpermeability in a rat model of hemorrhagic shock

**DOI:** 10.1038/cddiscovery.2015.42

**Published:** 2015-11-09

**Authors:** B Tharakan, SI McNeal, FA Hunter, DA Sawant, WR Smythe, EW Childs

**Affiliations:** 1 Department of Surgery, Texas A&M University Health Science Center College of Medicine, Baylor Scott and White Healthcare, Temple, TX, USA; 2 Department of Surgery, Morehouse School of Medicine, Atlanta, GA, USA; 3 School of Anatomical Science, Alderson Broaddus University, Philippi, WV, USA; 4 AVIA Health Innovation, Chicago, IL, USA

## Abstract

Following hemorrhagic shock (HS), vascular hyperpermeability, that is, the leakage of fluid, nutrients and proteins into the extravascular space occurs primarily due to the disruption of the endothelial cell–cell adherens junctional complex. Studies from our laboratory demonstrate that activation of the mitochondria-mediated ‘intrinsic’ apoptotic signaling cascade has a significant role in modulating HS-induced hyperpermeability. Here we report the novel use of recombinant Bcl-xL, an anti-apoptotic protein, to control HS-induced vascular hyperpermeability. Our results corroborate involvement of vascular hyperpermeability and apoptotic signaling. HS (the mean arterial pressure (MAP) was reduced to 40 mm Hg for 60 min followed by resuscitation to 90 mm Hg for 60 min) in rats resulted in vascular hyperpermeability as determined by intravital microscopy. Treatment of Bcl-xL (2.5 *µ*g/ml of rat blood in non-lipid cationic polymer, i.v.) before, during and even after HS attenuated or reversed HS-induced vascular hyperpermeability significantly (*P*<0.05). Conversely, treatment using Bcl-xL inhibitors, 2-methoxy antimycin (2-OMeAA) and ABT 737, significantly increased vascular hyperpermeability compared with sham (*P*<0.05). Bcl-xL treatment also decreased the amount of fluid volume required to maintain a MAP of 90 mm Hg during resuscitation (*P*<0.05). HS resulted in an increased mitochondrial reactive oxygen species formation, reduction of Δ*Ψ*m, mitochondrial release of cytochrome *c* and significant activation of caspase-3 (*P*<0.05). All of these effects were significantly inhibited by Bcl-xL pre-treatment (*P*<0.05). Our results show that recombinant Bcl-xL is effective against HS-induced vascular hyperpermeability that appears to be mediated through the preservation of Δ*Ψ*m and subsequent prevention of caspase-3 activation.

## Introduction

One of the clinical manifestations of hemorrhagic shock (HS) is disruption of the vascular endothelial cell barrier, which leads to the leakage of fluid, nutrients and proteins into the extravascular space.^1^ Leakage of fluids into third spaces during HS leads to lowered cardiac output and hypoperfusion of vital organs. If left untreated, the organs will begin to fail. HS accounts for nearly 30% of the deaths associated with traumatic injury.^2^ HS following trauma is shown to induce reactive oxygen species (ROS) formation and activation apoptotic signaling in the endothelium.^3–6^ Our laboratory is interested in the involvement of apoptotic signaling as a key regulator of vascular permeability following HS.^7–9^ We have shown that infusion of the pro-apoptotic protein BAK induces vascular hyperpermeability in rats at comparable levels to a model of HS.^10^ Further studies were undertaken that shows Bcl-xL abrogates the paracellular hyperpermeability initiated by tumor necrosis factor *α* (TNF*α*) *in vitro.*^8^ From these data, we hypothesized that Bcl-xL can be used as a therapeutic during HS. In this study, we use a novel *in vivo* protein transference method^9,11^ to introduce recombinant mouse Bcl-xL (rBcl-xL) into exchange vessels and monitor change in HS-induced vascular hyperpermeability. We also monitor mitochondrial ROS formation and membrane potential *in vivo*, utilizing dihydrorhodamine 123 and JC-1 (5,5’6, 6’-tetrachloro-1,1’,3,3’tetraethylbenzimidazolyl-carbocyanine iodide), respectively, and investigate the release of cytochrome *c*, and activation of caspase-3. We also perform *in vitro* parallel studies in microvascular endothelial cells. Our results demonstrate that exogenous delivery of rBcl-xL effectively prevents HS-induced vascular hyperpermeability in rats and is mediated via inhibition of the intrinsic apoptotic signaling cascade.

Apoptosis classically follows two pathways: the extrinsic (external stimuli) and the intrinsic (internal stimuli).^12,13^ The extrinsic pathway of apoptosis is initiated by binding the extracellular receptors in the TNF receptor superfamily, commonly referred to as the death receptors, for example, Fas, TNF*α* and TNF-related apoptosis-inducing ligand (TRAIL).^13,14^ The intrinsic pathway is activated by internal cellular damage, for example, hypoxia, ischemia and ROS generation.^15–18^ The result of activation is the release of cytochrome *c* from the mitochondria forming the apoptosome and activating caspase 9. Activated mitochondria release cytochrome *c*, apoptosis-inducing factor and second mitochondrial-derived activator of caspases, all of which are regulated by members of the Bcl-2 family of proteins (14, 51). The Bcl-2 family consists of both anti-apoptotic (Bcl-2, Bcl-xL and mcl-1) and pro-apoptotic (BAK and BAX) members^15,19,20^ Therefore, mitochondrial ROS is believed to have an important role in mitochondria-mediated intrinsic apoptotic signaling.

## Results

### rBcl-xL treatment attenuated vascular hyperpermeability following HS in rats

**Figure 1:**
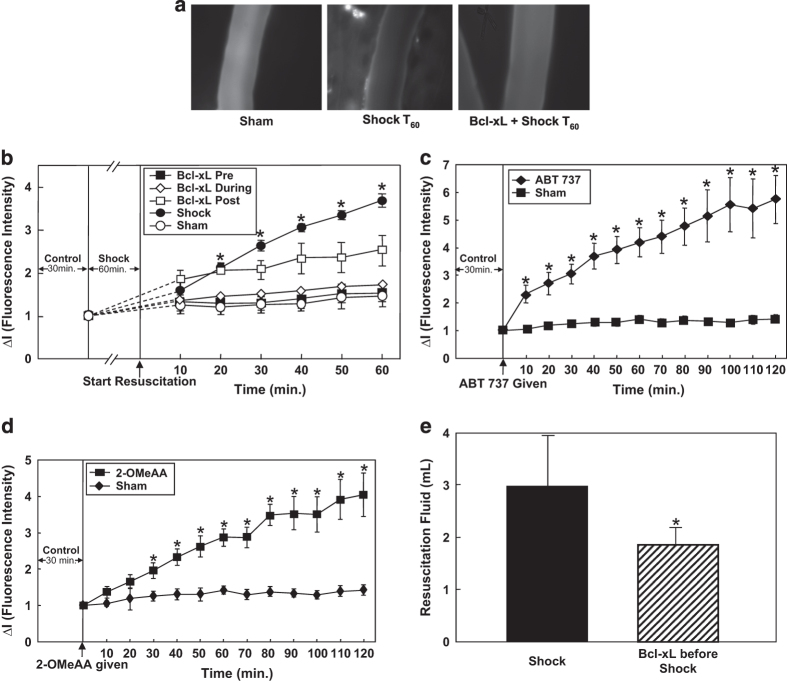
rBcl-xL prevents HS-induced vascular hyperpermeability in rat mesentery. (**a**) Representative mesenteric post-capillary venules from sham, HS for 1 h (*T*_60_) and Bcl-xL pre-treatment in shock are shown (**b–d**) FITC–albumin extravasation into the extravascular space is measured following HS. (**b**) All treatment arms of rBcl-xL are not significantly different from Sham. FITC–albumin extravasation is significantly high and time-dependent, following HS (*P*<0.05; *n*=5). (**c**) The Bcl-xL inhibitor 2-OMeAA induces vascular hyperpermeability in a similar manner as HS (*P*<0.05; *n*=5). (**d**) The BH3 inhibitor ABT 737 induces vascular hyperpermeability in a similar manner as HS (*P*<0.05; *n*=5). (**e**) rBcl-xL treatment reduced the amount of resuscitation fluid required to achieve a MAP of 40 mm Hg. * Significant difference *versus* hemorrhagic shock group and Bcl-xL plus hemorrhagic shock groups (*P*<0.05).

We have established that HS induces vascular hyperpermeability in mesenteric post-capillary venules. Here we observe a significant increase in extravasation of fluorescein isothiocyanate-bovine albumin (FITC–albumin) into the extravascular space *versus* the sham-operated animals (*P*<0.05; *n*=5; Figure 1a). When we inject rBcl-xL, an anti-apoptotic protein, before, during and after the HS period, we observe a significant attenuation of hyperpermeability compared with the HS group without Bcl-xL treatment (*P*<0.05; *n*=5; Figure 1b). Change in fluorescence intensity (*ΔI*) in the rBcl-xL treatment groups increases depending on the time in which rBcl-xL is injected. Although the *ΔI* increases, none of the rBcl-xL treatment groups are statistically different from sham. Exogenous administration of anti-apoptotic protein Bcl-xL reduces the mesenteric post-capillary venule hyperpermeability associated with HS.

### Bcl-xL transfection is sufficient to increase vascular permeability *in vivo*

To determine whether the reduction of vascular hyperpermeability is due to the transfection of Bcl-xL, we utilize the Bcl-xL/Bcl-2 inhibitors 2-OMeAA and ABT 737. Both treatment groups show significant increases in vascular hyperpermeability compared with the untreated sham group (Figures 1c and d). The FITC–albumin fluorescence intensity in the 2-OMeAA-treated group shows a time-dependent increase starting at 30 min (*P*<0.05; Figure 1c). The FITC–albumin fluorescence intensity in the ABT 737-treated group shows a time-dependent increase starting at 10 min (*P*<0.05; Figure 1d).

### Bcl-xL transfection reduces the amount of resuscitation fluid post HS

To better understand the clinical significance of Bcl-xL attenuation of HS-induced vascular permeability, we evaluated the volume of fluid necessary to resuscitate our animals following HS. Bcl-xL significantly reduced the fluid requirement compared with the HS-alone group (*P*<0.05, Figure 1e).

### Recombinant Bcl-xL attenuates mitochondrial ROS formation *in vivo*

Bcl-xL prevents apoptosis by sequestering pro-caspases or preventing the release of mitochondrial apoptogenic factors. In contrast, the pro-apoptotic members of this family, such as BAK, trigger the release of mitochondrial apoptogenic factors into the cytoplasm by acting on the mitochondrial transition pore (MTP). We also reported that this increase in endothelial ROS following HS is associated with an increase in microvascular hyperpermeability. We have demonstrated *in vitro* that ROS activation of the mitochondria has been shown to result in the release of apoptogenic factors such as cytochrome *c*.^21^

**Figure 2:**
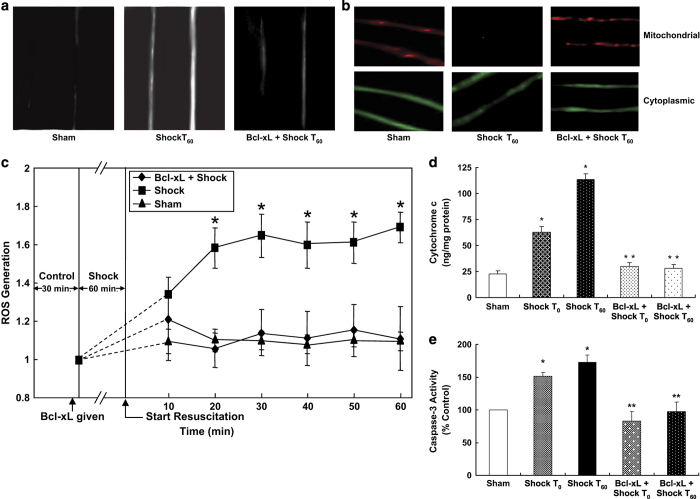
rBcl-xL prevents HS-induced mitochondrial ROS formation and cytochrome *c* release in the rat mesentery. (**a**) Representative images of mesenteric post-capillary venules of sham, Shock *T*_60_ and rBcl-xL treatment 10 min before HS (Bcl-xL+Shock *T*_60_) are shown. (**b**) Change in fluorescence intensity of mesenteric post-capillary venules of sham, shock and Bcl-xL+Shock are shown. Significantly increased ROS formation is observed following hemorrhagic shock compared with the sham group (*P*<0.05; *n*=5), whereas rBcl-xL treatment maintains sham levels of ROS. (**c**) rBcl-xL prevents hemorrhagic shock-induced decrease in Δ*Ψ*_m_. In sham, the JC-1 fluoresces red inside the mitochondria (top left panel) and the cytoplasm green (bottom left panel) indicating Δ*Ψ*_m_. Shock *T*_60_ demonstrates a loss Δ*Ψ*_m_ (top middle panel) Bcl-xL +Shock *T*_60_ treatment maintains Δ*Ψ*_m_ (top right panel). (**d**) Bcl-xL inhibits HS-induced cytochrome *c* release in the mesenteric vasculature. Cytosolic cytochrome *c* levels increase significantly at 0 and 60 min after resuscitation compared with sham. rBcl-xL inhibited hemorrhagic shock-induced increase in cytochrome *c* levels significantly (*P<0.05 *versus* sham group; **P<0.05 *versus* shock group). (**e**) rBcl-xL inhibits hemorrhagic shock-induced caspase-3 activation in the mesenteric vasculature. Caspase-3 activity increases significantly after shock at 0 and 60 min after resuscitation compared with sham. Bcl-xL inhibited hemorrhagic shock-induced caspase-3 activation significantly (*P<0.05 *versus* sham group; *n*=5; **P<0.05 *versus* shock group).

Utilizing the ROS-specific dye DHR 123, we took images of a sham rat’s mesenteric post-capillary venule demonstrating minimal ROS formation (Figure 2a, left panel). Following 60 min of HS and 60 min of resuscitation (*T*_60_) we observe a significant increase in ROS formation (Figure 2a, middle panel). rBcl-xL pre-treatment attenuates ROS formation and the *ΔI* is not dissimilar to Sham *ΔI* (Figure 2a, right panel). To further assess the role of Bcl-xL we assayed the amount of ROS produced by exogenous delivery of rBcl-xL during HS. In rats, HS results in a significant increase in mitochondrial ROS formation compared with the sham-control group (*P*<0.05; Figure 2b). Bcl-xL pre-treatment, that is, 10 min before the HS period results in a significant attenuation of ROS formation compared with the HS group that did not receive rBcl-xL (*P*<0.05; Figure 2b).

### Recombinant Bcl-xL decreased mitochondrial transmembrane depolarization *in vivo*

To determine the role that exogenous Bcl-xL has on the Δ*Ψ*_m_, we utilized JC-1 as described previously.^10,22^ A mesenteric post-capillary venule from a sham-control rat demonstrates both green (cytosolic) and red (mitochondrial) fluorescence in the vascular endothelial cells (Figure 2c, left panels). Following HS and 60 min of resuscitation, there was a decrease in red (mitochondrial) fluorescence indicating the loss of Δ*Ψ*_m_ (Figure 2c, middle panels). Bcl-xL pre-treatment prevented the loss of Δ*Ψ*_m_ evident by the increase in red fluorescence compared with the HS group without Bcl-xL treatment (Figure 2c, right panels).

### Recombinant Bcl-xL decreased mitochondrial cytochrome *c* release

Release of cytochrome *c* from mitochondria into the cytosol through the MTP is the major route of caspase activation. Cytoplasmic cytochrome *c* leads to the release of the apoptosome assembly from apoptotic protease-activating factor-1 (Apaf-1), ATP and procaspase-9, which ultimately activates the effector caspase.^23^ Thus, alterations in mitochondrial membrane integrity via pro-apoptotic factors and the subsequent release of cytochrome *c* are the key components in the apoptotic signaling cascade. Previous studies from our laboratory implicated the mitochondria of endothelial cells as a major producer of ROS following HS.^24^ Release of cytochrome *c* from the mitochondria into the cytosol following the opening of the MTP has been reported to be the key event in apoptosis induced by various stimuli. In our data, cytoplasmic cytochrome *c* levels are elevated following HS at *T*_0_ (60 min of HS and 0 min resuscitation) and at *T*_60_ (60 min of HS and 60 min of resuscitation) compared with sham-control group (*P*<0.05). The rBcl-xL-pre-treated HS group (*T*_0_ and *T*_60_) shows significantly lower levels of cytoplasmic cytochrome *c* compared with the HS group without rBcl-xL treatment (*P*<0.05; Figure 2d).

### Recombinant Bcl-xL attenuated caspase-3 activation

HS groups *T*_0_ and *T*_60_ show significant increase in caspase-3 activity compared with sham-control group (*P*<0.05; Figure 2e). rBcl-xL treatment before HS shows significantly lower caspase-3 activity compared with HS without Bcl-xL pre-treatment (*P*<0.05; Figure 2e).

### *In vitro* studies

#### Bcl-xL prevents BAK-induced monolayer hyperpermeability of microvascular endothelial cell monolayers

**Figure 3:**
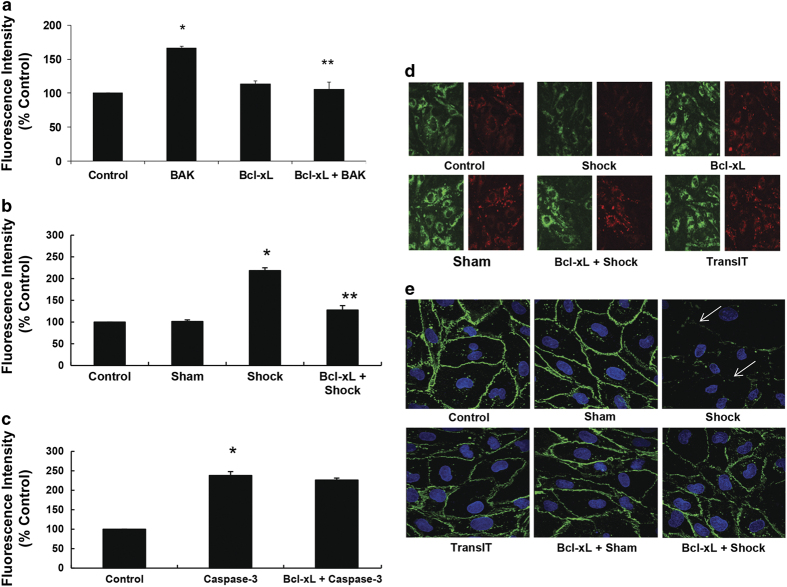
rBcl-xL attenuates BAK-induced hyperpermeability in RLMEC monolayers. (**a**) Change in permeability is expressed as percentage of the basal fluorescence. BAK transfection induced hyperpermeability in the monolayer compared with control (*P<0.05; *n*=5). Bcl-xL pre-treatment in BAK-transfected cells showed decrease in FITC–albumin fluorescence compared with untreated cells (**P<0.05; *n*=5). (**b**) rBcl-xL attenuates shock serum-induced hyperpermeability in RLMEC monolayers. The fluorescence intensity is significantly higher in the shock serum-treated group compared with the control group or sham serum-treated group showing an increase in permeability of the monolayer. The monolayers pre-treated with rBcl-xL attenuated the shock serum-induced hyperpermeability significantly. (**c**) rBcl-xL acts upstream of caspase-3-induced hyperpermeability in rat lung microvascular endothelial cell monolayers. The FITC–albumin fluorescence intensity is significantly higher in the active caspase-3-transferred group compared with the control group (*P*<0.05), suggesting an increase in permeability of FITC–albumin across the endothelial cell monolayer. The monolayers pre-treated with rBcl-xL show no significant decrease in hyperpermeability. (**d**) Bcl-xL protects mitochondrial membrane integrity in RLMECs. Fluorescence microscopy images of the mitochondrial membrane potential indicator JC-1 in its monomeric (green) and dimeric (red) forms are shown. Treatment of HS serum leads to the loss of Δ*Ψ*_m_, showing predominantly monomeric forms. Bcl-xL treatment prevents the collapse of mitochondrial membrane potential evidenced by the restoration of dimeric form-red fluorescence. (**e**) rBcl-xL protects endothelial adherens junction complex from hemorrhagic serum-induced disruption in rat lung microvascular endothelial cell monolayers. Shock serum treatment shows disruption of adherens junction evident from diffused fluorescence of *β*-catenin as well as intercellular gap formations. The cells pre-treated with Bcl-xL did not show a visible change in *β*-catenin distribution at the adherens junction indicating intact barrier functions. Bcl-xL transference or TransIT alone treatment showed no visible change in *β*-catenin distribution compared with untreated control cells.

Rat lung microvascular endothelial cell (RLMEC) monolayers were used to evaluate vascular hyperpermeability. The FITC–albumin fluorescence intensity is significantly higher in the BAK peptide-transfected group compared with the control group (*P*<0.05; Figure 3a), consistent with hyperpermeability we have observed in previous studies following activation of apoptosis.^8,25^ We incubated RLMEC with serum from HS rats followed by rBcl-xL treatment and observed significant reduction of hyperpermeability compared with HS alone (*P*<0.05; Figure 3b). The monolayers pre-treated with rBcl-xL also attenuated the caspase-3-induced vascular hyperpermeability significantly (*P*<0.05; Figure 3c).

#### Bcl-xL protects mitochondrial transmembrane potential of microvascular endothelial cell monolayers

RLMEC monolayers from the untreated control group show red fluorescence indicating J-aggregate formation and intact mitochondria (Figure 3d, top left). Sham serum, Bcl-xL or TransIT-alone treatments show no visible change in mitochondrial transmembrane potential compared with untreated control monolayers (Figure 3d). Following HS serum treatment, there is a decrease in mitochondrial (red) fluorescence indicating the loss of MTP. RLMEC monolayers treated with HS serum and rBcl-xL maintain red fluorescence, which indicates the preservation of the MTP (Figure 3d, middle panel).

#### Bcl-xL prevents adherens junction damage in microvascular endothelial cells

RLMEC from the untreated control group and the sham serum-treated group displays continuous distribution of FITC-*β*-catenin at the adherens junctional complex (Figure 3e). Following HS serum treatment, the adherens junctions are disrupted as evidenced by diffused punctuate distribution of *β*-catenin and formation of intercellular gaps (Figure 3e, top right). HS serum-treated cells pre-treated with rBcl-xL prevents the disruption of *β*-catenin at the adherens junction (Figure 3e, bottom right).

## Discussion

Death receptors are known to be activated during ischemia-reperfusion injury in the microvasculature. In particular, the TNF and TRAIL receptors become activated and initiate both death and pro-survival signaling.^26^ By definition, our experimental model of HS produces a period of ischemia followed by a reperfusion event. Using this model, our laboratory has shown a direct correlation between apoptotic signaling and HS-induced vascular hyperpermeability.^10^ Specifically, transfection of the pro-apoptotic Bcl-2 family member BAK is sufficient to initiate hyperpermeability of the mesenteric vasculature in rats following HS.^8^ Here we report that exogenous delivery of the rBcl-xL protein, a member of the Bcl-2 family of proteins, attenuates vascular hyperpermeability, mitochondrial depolarization, ROS production and the activation of caspase-3 in rats following experimental HS.

All members of the Bcl-2 family share one or more Bcl-2 homology (BH) domains and are divided into two main groups according to whether they are pro- or anti-apoptotic. The pro-apoptotic members of the Bcl-2 family are subdivided according to whether they contain multiple BH domains or only the required BH3 domain. The pro-apoptotic protein BAK is a multidomain BH3 protein that induces loss of Δ*Ψ*_m_ via the mitochondrial permeability transition pore.^27^ BAK and BAX oligomerization is important for the permeabilization of the mitochondrial outer membrane. In our study, rBcl-xL attenuated BAK-induced monolayer hyperpermeability and presumably in the mesenteric vasculature, given the significantly lower levels of detection of ROS and mitochondrial depolarization (see Figures 2b and c). Bcl-xL is known to antagonize the effects of pro-apoptotic BAK at the level of mitochondria, and the protection by Bcl-xL observed in this study may be attributed to its protective effect against BAK-induced permeabilization of mitochondrial membrane. Our recent studies have shown that BAK peptide induces vascular hyperpermeability in rats.^10^ Furthermore, collapse of Δ*Ψ*_m_, mitochondrial release of cytochrome *c* into the cytoplasm and the activation of caspase-3 occurs following BAK peptide treatment. We suspect that overexpression of an anti-apoptotic protein such as Bcl-xL is outcompeting BAK and BAX for their binding site, preventing their oligomerization and opening of the MTP.

Further support for apoptosis having a major role in vascular permeability was confirmed by the observation that the specific Bcl-xL inhibitor and a pan-BH3 inhibitor, 2-OMeAA and ABT 737, respectively, induced vascular hyperpermeability following Bcl-xL transcription in the rat (see Figures 1c and d). We demonstrate the therapeutic usefulness of Bcl-xL in HS by observing that administration of rBcl-xL reduces the amount of fluid required for resuscitation following HS, that is, less vascular permeability (see Figure 1e). Thus, our study shows the effectiveness of Bcl-xL treatment against HS-induced microvascular hyperpermeability by inhibition of the intrinsic apoptotic signaling cascade.

The effective use of an endogenous anti-apoptotic protein Bcl-xL has high significance in therapeutic intervention against vascular hyperpermeability. Exogenous administration of Bcl-xL has been attempted previously. It has been shown that systemic delivery of recombinant Bcl-xL fusion protein containing the TAT protein transduction domain attenuated neonatal brain damage following hypoxic ischemia in 7-day-old rats, and the TAT-Bcl-xL has been shown to inhibit caspase-3 and -9 activity.^28^ These data suggest that Bcl-xL is effective against ischemia-induced apoptotic cell death. The protective effect of Bcl-xL observed in an acute condition such as HS-induced vascular hyperpermeability may be due to its protective effect against adherens junction damage before cell death. We have observed this effect in other studies, where we observed *β*-catenin redistribution from cell–cell junctions into perinuclear aggregations following active caspase-3 transference.^9^ These data suggest that during HS the vascular endothelium undergoes apoptosis, which leads to detachment of endothelial cells. Other laboratories report that endothelial-derived microparticles and other common endothelial cell surface markers can be used as biomarkers for acute and chronic pathology.^29,30^ We believe that the endothelium is a sentinel that is sensitive to physiologic changes and as such are susceptible to hyperpermeability following most insults. What we have reported above demonstrates the ability of Bcl-xL to reduce vascular hyperpermeability before, during and after HS (see Figure 1b). Reduction of vascular hyperpermeability post HS by an anti-apoptotic protein suggests that apoptosis is ‘pre-primed’, or continuously occurring, in the epithelium. However, what remains to be explored is how these cells recover following insult without total loss of barrier function. Some areas to explore are in shear stress, cell surface receptor trafficking and cell–cell interaction during HS.

In conclusion, our results demonstrate that rBcl-xL protein effectively prevents HS-induced vascular hyperpermeability, possibly by inhibiting HS-induced activation of the intrinsic apoptotic signaling cascade. Our study has considerable therapeutic potential against HS-induced vascular hyperpermeability as well as in other types of trauma where activation of the apoptotic signaling cascade is known.

## Materials and Methods

### Animals

Adult male Sprague–Dawley rats (250–275 g; Charles River Laboratories, Wilmington, MA, USA) were maintained at Texas A&M Health Science Center College of Medicine and Scott and White Memorial Hospital animal facility on a 12 : 12-h dark/light cycle, with free access to food and water. The room temperature was maintained at 25±2 °C. The surgical and experimental procedures used in this study were conducted after approval by the Institutional Animal Care and Use Committee. The facility is approved by the American Association for Accreditation of Laboratory Animal Care in accordance with the National Institutes of Health guidelines.

### Reagents

The test solute used for the vascular hyperpermeability measurements was FITC–albumin obtained from Sigma (St. Louis, MO, USA). The test solution was prepared by dissolving the FITC–albumin in 50 mg/kg saline. JC-1 (5,5’,6,6’-tetrachloro-1,1’,3,3’-tetraethylbenzimidazolylcarbocyanine iodide) was obtained from Cell Technology Inc. (Mountain View, CA, USA). The JC-1 reagent was prepared by reconstituting the lyophilized reagent with DMSO to obtain a 100× stock solution. Immediately before the experiments, the 100× solution was diluted 1 : 100 in 1× JC-1 assay buffer. DMSO at this dilution does not have a significant effect on vascular hyperpermeability. rBcl-xL protein obtained from R&D Systems (Minneapolis, MN, USA) was dissolved in DMSO. Bcl-xL was mixed with TransIT (a non-lipid cationic polymer, Mirus Bio-Corporation, Madison, WI, USA) for a final concentration of 2.5 *μ*g/ml (Bcl-xL) and 10 *μ*l/ml of TransIT based on rat’s blood volume. Bcl-xL inhibitor 2-OMeAA was obtained from Sigma. The Bcl-xL inhibitor ABT 737 was a generous gift from Abbot Laboratories (Abbott Park, IL, USA).

### *In vivo* studies

#### HS and intravital microscopy

Male Sprague–Dawley rats were fasted for 18 h before each experiment to minimize the amount of undigested food particles in the intestine. The animals were anesthetized by a single injection of 50% urethane (1.5 g/kg) given intramuscularly. Polyethylene tubing (PE-50, 0.58 mm ID) was placed into the right internal jugular vein for drug administration and fluid resuscitation and the right carotid artery for blood withdrawal, respectively. The mean arterial pressure (MAP) was monitored continuously using a PE-50 (0.58 mm ID) cannula placed in the left femoral artery connected to a blood pressure analyzer (Dig-Med, BPA 400A, Micromed, Louisville, KY, USA). A midline laparotomy incision was made, and the rats were placed in a lateral decubitus position on a Plexiglas platform mounted to an intravital upright microscope (Nikon E600, Nikon, Tokyo, Japan). A section of the mesentery from the proximal ileum was exposed and maintained at 37 °C. The mesentery was superfused with normal saline at 2 ml/min via cannula while covered with plastic wrap to reduce evaporation. Post-capillary venules with diameters of 20–35 *μ*m were selected for study utilizing intravital microscope (Nikon Instruments Inc., Natick, MA, USA). The same venule was studied throughout each experiment. Images were obtained with a Photometric Cascade Camera (Roper Scientific, Tucson, AZ, USA), projected onto a computer monitor and captured digitally on computer disc. Data analysis was performed using MetaMorph 4.5/4.6 (Universal Imaging Corp, Downingtown, PA, USA).^31,32^ The animals were allowed to equilibrate for 30 min before each experiment. This time period allowed the animals to recover from surgical manipulation. Following the equilibration period, the animals were given FITC–albumin (50 mg/kg) and baseline-integrated optical intensities were obtained intra- and extravascularly (two sites, same computed areas, the mean values were used). To produce HS, the MAP was decreased to 40 mm Hg by withdrawing blood from the right carotid artery into a heparinized syringe. To obtain this level of HS requires the removal of ~50–60% of the animal’s blood volume (level IV shock). After HS, the shed blood plus normal saline was reinfused within 5 min to maintain a MAP at or above 90 mm Hg. Mesenteric post-capillary venules in a transilluminated segment of the small intestine were examined to quantitate changes in the FITC–albumin flux. Parameters were recorded immediately after the shock period and at every 10 min interval for 60 min.

#### Vascular hyperpermeability *in vivo*

The rats were divided into the following groups: sham (*n*=5), shock (*n*=5) and HS group plus Bcl-xL (Bcl-xL pre; *n*=5), given 10 min before the shock period. HS group plus Bcl-xL (Bcl-xL during; *n*=5) given 10 min into the shock period. HS group plus Bcl-xL (Bcl-xL post; *n*=5) given 10 min after the shock period. TransIT (10 *μ*l/ml) concentration was determined from previous published data in intact microvessels.^11^ The cocktail was prepared by mixing 200 *μ*l of serum-free media with 160 *μ*l of TransIT at room temperature for 15 min. Bcl-xL protein was mixed with the prepared TransIT solution and was allowed to equilibrate at room temperature for an additional 15 min. The cocktail was injected intravenously into the internal jugular vein. After 60 min of peptide delivery, the animals were injected intravenously with FITC–albumin (50 mg/kg) to quantitate changes in the albumin flux, as described above. The FITC–albumin flux was measured by determining the changes in integrated optical intensity. The changes in optical intensity were determined in the extravascular space relative to the intensity inside the lumen. The following formula was used to calculate change in optical intensity; *ΔI*=1−(*I*i−*I*o/*I*i), where *ΔI* is the change in fluorescence intensity, *I*i is fluorescence intensity inside the vessel and *I*o is fluorescence intensity outside the vessel.^31,32^

Additional experiments were performed to determine whether the attenuation of vascular hyperpermeability by Bcl-xL correlated with a reduction in volume necessary to maintain a MAP at or above 90 mm Hg.

In another set of experiments, Bcl-xL inhibitors 2-OMeAA (6 *μ*g/ml) and ABT 737 (40 *μ*M) were tested for their effect on vascular hyperpermeability. The experimental groups consisted of sham-control (*n*=5), 2-OMeAA (*n*=5) and ABT 737 (*n*=5). The animals were injected intravenously with FITC–albumin (50 mg/kg) during the equilibration period, and the FITC–albumin flux was again measured by determining the changes in integrated optical intensity.

### *In vitro* studies

#### Mitochondrial ROS formation *in vivo*

The rats were divided into the following groups: sham-control (*n*=5), HS (*n*=5) and Bcl-xL plus HS (*n*=5). HS was induced as described above. Visualization and quantification of ROS from the mesenteric post-capillary venules was performed using dihydrorhodamine 123 (DHR 123; 50 mg/kg) using intravital microscopy. Images of the mesenteric venules were obtained before the shock period and at 10, 20, 30, 40, 50 and 60 min into resuscitation. To determine relative expression of ROS, the fluorescent intensity was measured in two areas along the vessel using MetaMorph 4.5/4.6 (Universal Imaging Corp.). Values of DHR 123 fluorescence were expressed as change in intensity over time *versus* baseline values.

#### Mitochondrial transmembrane potential *in vivo*

The rats were divided into the following groups: sham-control (*n*=5), HS group (*n*=5) and HS group that received Bcl-xL (2.5 *µ*g/ml). Bcl-xL was given 10 min before the shock period. The mesenteric vasculature was superfused with JC-1 reagent (1 :  100) to measure changes in mitochondrial transmembrane potential utilizing intravital microscopy as previously described in our laboratory.^22,33^ The JC-1 reagent was superfused over the exposed mesenteric vessels at a volume of 300 *μ*l into a bath of 2 ml of saline. The JC-1 rapidly diffused into the vasculature and was detected in the endothelial cells.

### *In vitro* studies

#### Cytosolic cytochrome *c* levels

The rats were divided into the following groups: sham group (*n*=5), HS for 60 min tissue taken at the beginning of resuscitation (HS *T*_0_; *n*=5), HS for 60 min tissue taken at the end of 60 min resuscitation (HS *T*_60_; *n*=5), a HS *T*_0_ resuscitation plus Bcl-xL group (*n*=5) and a HS *T*_60_ resuscitation plus Bcl-xL group (*n*=5). The cytosolic cytochrome *c* levels were estimated using a cytochrome *c* ELISA kit (R&D systems, Minneapolis, MN, USA). The mesenteric vessels were dissected from the rat, weighed and homogenized in a cold preparation buffer (10 mM Tris-HCl pH=7.5, 0.3 M sucrose, 10 *μ*M Apoptinin, 10 *μ*M Pepstatin, 10 *μ*M Leupeptin and 1 mM PMSF). The tissue homogenates were centrifuged (10 000 r.p.m. for 60 min at 4 °C), and the supernatant (cytosolic fraction) was collected and subjected to protein estimation. The cytosolic cytochrome *c* levels were estimated using a cytochrome *c* ELISA kit (R&D systems). In brief, the samples were treated with a conjugate reagent (horseradish peroxidase-conjugated anti-cytochrome *c* polyclonal antibody), transferred to microwell strips coated with anti-cytochrome *c* antibody and incubated for 60 min at room temperature. The well contents were discarded and the wells were washed using a wash solution. The samples were then treated with a peroxidase substrate reagent and incubated for 15 min at room temperature. Following the addition of a stop solution, the optical density of each well was measured at 450 nm. A serial dilution of cytochrome *c* calibrator was subjected to the assay along with the samples and the values were plotted. The concentration of cytochrome *c* was calibrated from the standard curve.

#### Casapse-3 activity

The rats were divided into the following groups: sham group (*n*=5), HS for 60 min tissue taken at the beginning of resuscitation HS *T*_0_ (*n*=5), HS for 60 min tissue taken at the end of 60 min resuscitation period HS *T*_60_ (*n*=5), a HS *T*_0_ resuscitation plus Bcl-xL group (*n*=5) and a HS *T*_60_ resuscitation plus Bcl-xL group (*n*=5). The mesenteric microvessels harvested from the animals were homogenized in caspase-3 sample lysis buffer provided in the caspase-3 fluorometric assay kit (Calbiochem, La Jolla, CA, USA). The homogenates were centrifuged at 500×*g*, and the resulting supernatant was used for protein estimation and caspase-3 assays. Active Caspase-3 cleaves after aspartate residues in a particular peptide sequence (DEVD). The DEVD substrate was labeled with a fluorescent molecule, 7-amino-4-trifluoromethyl coumarin. The lysates were treated with the substrate conjugate, and the resulting fluorescence was measured in a fluorescent plate reader at 400 nm/505 nm.

#### Monolayer permeability *in vitro*

For some studies, blood was collected previously from a group of HS rats, or sham rats, and centrifuged at 6000 r.p.m. for 20 min to obtain serum. RLMECs obtained from VEC Technologies Inc. (Rensselaer, NY, USA) were grown as monolayers in transwell plates (Corning Life Sciences, Lowell, MA, USA) in complete MCDB-3 media. Sixty minutes before the experiments, the monolayers were incubated in fresh media without phenol red dye. The monolayers were transfected with Bcl-xL (2.5 *μ*g/ml) using TransIT for 60 min. The cells were then treated with the pro-apoptotic BAK peptide, HS serum (*T*_60_) or caspase-3. Bcl-xL and BAK (5 *μ*g/ml) were exposed to TransIT (10 *μ*l/ml) for 15 min before exposure to the cells FITC–albumin (5 mg/ml) was added to the luminal (upper) chamber of the transwell and were allowed to equilibrate for 30 min. Untreated monolayers were used as controls. Previous studies from our laboratory have shown that the transfection medium alone does not induce hyperpermeability in the monolayer. The samples (100 *μ*l) collected from the abluminal (lower) chambers were analyzed for FITC fluorescent intensity using a fluorometric plate reader at excitation 494 and 520 nM. The data were calculated as percentage of the control (basal) values.

#### Mitochondrial transmembrane potential *in vitro*

Mitochondrial transmembrane potential (Δ*Ψ*_m_) was studied using a cationic fluorescent indicator JC-1 described above. To determine change in Δ*Ψ*_m_, RLMECs were grown on fibronectin-coated cover glass bottom dishes for 24 h, exposed to media without phenol red for 60 min followed by transfection of Bcl-xL (*μ*M). Bcl-xL-treated or untreated cells were exposed to sham or shock serum for 60 min. Bcl-xL (2.5 *μ*g/ml) was prepared as described above, and transfections were performed for 60 min as described above. Untreated cells served as controls. The cells were incubated with JC-1 for 15 min at 37 °C, washed in a JC-1 wash buffer and observed immediately under a confocal (Olympus Fluoview, Center Valley, PA, USA) microscope for visualization of green and red fluorescence.

#### Endothelial cell adherens junctions

RLMECs were grown on fibronectin-coated 20-mm dishes in complete MCDB-3 media for 24 h. Sixty minutes before the start of experiments, the cells were exposed to low-serum media. The cells were transfected with Bcl-xL (2.5 *μ*g/ml) for 60 min. The cells were then treated with HS serum for 60 min. Blood was collected previously from a group of HS rats or sham rats and centrifuged at 6000 r.p.m. for 20 min to obtain serum. Bcl-xL was exposed to TransIT (10 *μ*l/ml) for 15 min before exposure to the cells.^8,11^ Untreated, sham serum-treated, TransIT-alone-treated and Bcl-xL-alone-treated cells served as controls. The cells were washed in PBS, permeabilized with Triton X-100 and fixed with 4% paraformaldehyde. The cells were then washed in PBS, blocked with 2.5% BSA-PBS and exposed to a polyclonal antibody against *β*-catenin (Santa Cruz Biotechnology, Dallas, TX, USA) overnight at 4 °C. The cells were washed in PBS and exposed to an FITC-tagged ant-rabbit secondary antibody (Santa Cruz Biotechnology). The cells were washed, mounted in an antifade-DAPI mountant and visualized utilizing confocal microscopy.

### Statistical analysis

All values are expressed as mean±S.E.M. Statistical analysis was performed utilizing analysis of variance (ANOVA) followed by the Bonferroni’s post-test for multiple comparisons. A *P’*value of <0.05 was considered to indicate a significant difference. In vascular permeability studies, each experimental value was compared with the initial baseline value and expressed as the percentage change. This method decreases bias between animals because of hematocrit and changes in room lighting. For all treatments, we did the following analysis. We built a linear model with a time factor and a treatment factor to allow us to do a two-way ANOVA. We also accounted for random variation between rats, by adding in random effects terms. Our outcome of interest, however, was the difference between treatments at each separate time point (for example, between drug and sham, or between Bcl-xL and so on). This was assessed using pre-planned contrasts from the linear model. Multiple testing was corrected using the Bonferroni correction.

## Abbreviations

2-OMeAA: 2-methoxy antimycin
Δ*Ψ*_m_: mitochondrial transmembrane potential
Δ*I*: change in fluorescence intensity
AFC: 7-amino-4-trifluoromethyl coumarin
Apaf-1: apoptotic protease-activating factor-1
BH: Bcl-2 homology
DHR 123: dihydrorhodamine 123
FITC: fluorescein isothiocyanate
HS: hemorrhagic shock
JC-1,5,5’6: 6’-tetrachloro-1,1’,3,3’tetraethylbenzimidazolyl-carbocyanine iodide
MAP: mean arterial pressure
MTP: mitochondrial transition pore
rBcl-xL: recombinant mouse Bcl-xL
RLMEC: rat lung microvascular endothelial cells
ROS: reactive oxygen species
smac: second mitochondrial-derived activator of caspases
*T*_60_: 60 min of HS and 60 min of resuscitation
*T*_0_: 60 min of HS and 0 min resuscitation
TNF*α*: tumor necrosis factor *α*
TRAIL: TNF-related apoptosis-inducing ligand.
